# A rare case of suture material obstructing the closure mechanism of a prosthetic aortic valve: a case report

**DOI:** 10.1186/1757-1626-2-9126

**Published:** 2009-12-02

**Authors:** Emaddin S Kidher, Ryan Perera, Christopher Rao, Syed M Rehman, Nilesh Sutaria, Thanos Athanasiou

**Affiliations:** 1 Department of Biosurgery and Surgical Technology, Imperial College London, St Mary's Hospital, London, W2 1NY, UK; 2 Department of Cardiology, Imperial College Healthcare NHS Trust, St Mary's Hospital, London, W2 1NY, UK

## Abstract

Prosthetic aortic valve dysfunction presenting as aortic regurgitation is a complication of mechanical valve replacement. We describe a case of late valve dysfunction caused by an annular suture of excessive length obstructing the closure mechanism of a bileaflet prosthetic valve.

We present this rare cause of valve dysfunction in an 80-year-old male patient who presented with haemolysis and dyspnoea. At the time of operation it was found that a long vertically positioned annular valve suture was interfering with the normal closure mechanism of one of the prosthetic leaflets causing eccentric regurgitation jets. These findings were misdiagnosed as paravalvular leaks on the preoperative transoesophageal echo. No paravalvular leak was identified intraoperatively. After removal of the responsible suture normal prosthetic valve function was restored.

Whilst early aortic valve dysfunction caused by suture material has previously been reported, this is the first report of suture material causing late dysfunction.

## Introduction

Prosthetic aortic valve dysfunction for any reason requires prompt diagnosis and therapy. It often presents as aortic regurgitation and is commonly caused by paravalvular leak (PVL). Suture-related early aortic prosthetic valve dysfunction has previously reported [1]. We report the case of late dysfunction caused by suture material.

## Case Presentation

An 80 year-old man underwent aortic valve replacement with a Carbomedics Sulzer size 21 mm mechanical prosthesis in 1999 for severe aortic stenosis. The patient made a successful recovery and had significant improvement in his initial symptoms without readmission for valve dysfunction.

**Figure 1:**
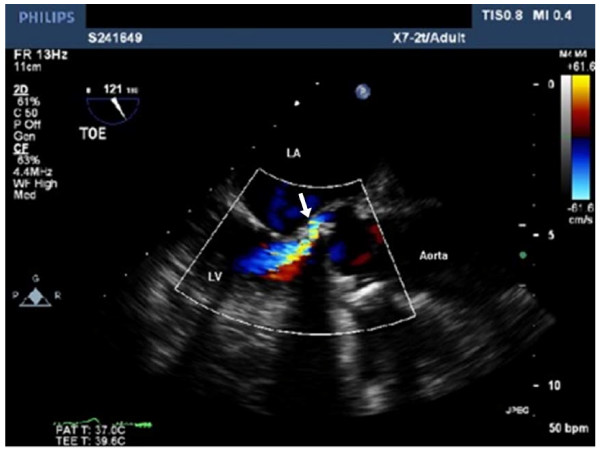
**Pre-operative transoesophageal echocardiogram in the Left Ventricular Outflow Tract view**. This appears to demonstrate an eccentric jet of aortic regurgitation (white arrow) from the posterior paraprosthetic margin of the aortic valve replacement.

In 2008, the patient represented with episodes of pulmonary oedema, worsening dyspnoea, and haemolytic anaemia requiring regular transfusion. Transoesophageal echocardiography (TOE) demonstrated moderate to severe aortic regurgitation with at least two eccentric jets, suggestive of PVL (Figure 1). Overall systolic function was preserved. Coronary angiogram revealed no significant disease. In view of the combination of significant PVL, dyspnoea and haemolytic anaemia, in a surgically fit patient, a decision was made to proceed surgically.

**Figure 2:**
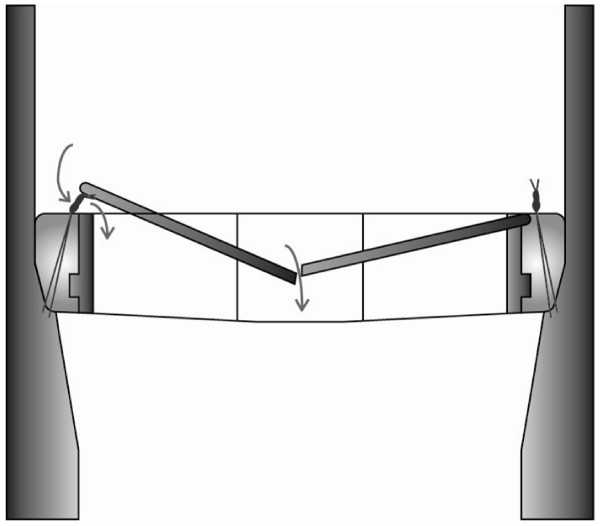
**Schematic representation of the patient's bileaflet mechanical aortic prosthetic valve during diastole**. This figure demonstrates the incomplete closure of one of the valve leaflet (because of the trapped suture material) and the associated regurgitant jet (arrows).

Despite thorough inspection at the time of operation, no PVL was found. However, a suture on the valve ring with almost 10 knots was found in an unusual position, vertical to the outflow of the valve within the area of the non-coronary sinus. This suture was obstructing one of the valve leaflets preventing complete closure (Figure 2). The stitch was removed and a pledgeted suture applied from the external part of the aorta to the aortic valve ring to prevent incomplete opposition between the annulus and sewing-ring. Intraoperative TOE confirmed normal valve function. The patient made a successful recovery and was discharged home within one week. A transthoracic echo (TTE) on the sixth postoperative day confirmed normal valve function.

## Discussion

Thrombosis, thromboembolism, endocarditis, pannus formation and PVL are recognised causes of non-structural valve dysfunction. Initially in our case the valve dysfunction was wrongly diagnosed as a PVL. PVL is the most common cause of non-structural prosthesis dysfunction, estimates of the prevalence of aortic PVL range widely from 6.0-47.6% [2,3], however only 0.6-1.7% require reoperation [4,5]. Causes of PVL may include; incomplete decalcification precluding fluid-tight suturing, inappropriate valve size, and excessive tension when tying a suture which can cause it to cut through the annular bed.

In the case we report valve dysfunction occurred as one valve leaflet was only able to partially close due to suture material trapped between the leaflet and the valve ring. This left two small areas of the valve open in diastole, allowing eccentric regurgitant jets (Figure 2). Our literature review found only one similar case report. Miyahara et al described a case which was treated by reducing the length of the suture and rotating the prosthesis [1]. In the Miyahara case, however, the condition was diagnosed very early in the post-operative period due to persistently low diastolic pressure and cardiac output. It is difficult to draw conclusions on the prevalence of this cause of valve dysfunction from the paucity of reported cases as there may be reluctance among cardiac surgeons to report cases which represent avoidable technical failure. Secondly, as we will discuss it is difficult to differentiate between suture obstruction and PVL preoperatively.

**Figure 3:**
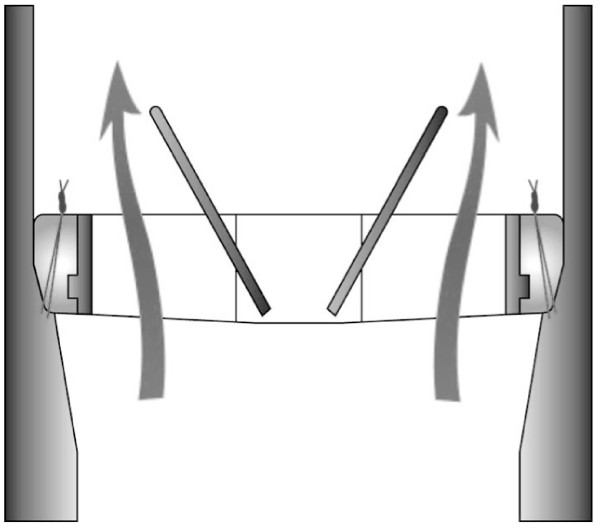
**Schematic representation of the bileaflet mechanical aortic prosthetic valve during systole**. This figure shows the normal length and position of suture material allowing normal function of the prosthesis.

Suturing techniques may play an important role in preventing the significant morbidity and mortality associated with this cause of prosthesis dysfunction. There is controversy associated with which sutures techniques are most effective for aortic valve replacement [6-9]. The conventional interrupted suture technique is considered more secure as each suture is independent from adjacent sutures, and consequently the risk that a loose suture loop could cause PVL is reduced. Continuous suturing techniques, however, are easier, reduce operative time [6] (including aortic cross-clamp time and cardio-pulmonary bypass time), reduce exposure of blood stream to plenty of foreign bodies (such as knots and pledgets) that may predispose the patient to thromboembolic and infective events, and prevent annular down-sizing. Hjelms et al suggested continuous sutures should only be used for stenotic lesions as the incidence of PVL is far lower when compared to patients with pure aortic insufficiency [8]. If an interrupted suture technique is used attention should be paid to the position and direction of suture knots, and the surgeon must ensure that sutures are cut short enough to prevent valve mechanism interference whilst remaining long enough to maintain knot strength and prevent slippage (Figure 3).

PVL is usually suspected from the clinical presentation and confirmed by echocardiography. Echocardiography with color Doppler facility and color flow mapping is the gold-standard tool for detecting, localizing and assessing the severity of PVL. Both TTE and TOE are useful tools for the assessment of prosthetic valve function. Whilst TOE is more invasive it is considered superior to TTE in assessing the severity of valve regurgitation [10]. In our case, we believe that PVL was wrongly diagnosed because of well recognised echocardiographic difficulties in differentiating between PVL and eccentric transvalvular regurgitation. Real-time 3D (RT3D) TOE is emerging as a promising tool in the management of PVL. RT3D TOE gives detailed en-face views of a paravalvular leak and surrounding structures. Such imaging could facilitate more accurate preoperative diagnosis of prosthesis dysfunction. Although the preoperative diagnosis was incorrect in the case we report, the correct diagnosis would have been unlikely to change the decision to operate as surgical removal of the obstructing suture would be the treatment of choice in this scenario. The emergence of percutaneous techniques for PVL closure will make correct preoperative diagnosis of the cause of valve dysfunction imperative in the future if inappropriate procedures are to be avoided.

In conclusion, whilst the incidence of this apparently rare cause of valve dysfunction is unknown it is important the cardiac surgeon is aware of suture-related valve dysfunction for several reasons. Firstly because judicious attention to suture and implantation technique can easily avoid the mortality associated with valve dysfunction and re-operation. Secondly because the surgeon must be conscious that suture-related valve dysfunction may mimic PVL when operating on patients diagnosed with prosthetic valve dysfunction. Finally, the emergence of catheter-based interventions makes accurate preoperative diagnosis imperative in order to select the most appropriate therapy.

## Consent

Written informed consent was obtained from the patient for publication of this case report and accompanying images. A copy of the written consent is available for review from the journal's Editor-in-Chief.

## Competing interests

The authors declare that they have no competing interests.

## Authors' contributions

All authors were major contributors in interpreting the clinical and radiological data of the patient. All authors were major contributors in writing the manuscript. All authors have approved the final version of the submitted manuscript and the corresponding author is able and willing to act as guarantor for this study
